# Ecology and diversity of culturable fungal species associated with soybean seedling diseases in the Midwestern United States

**DOI:** 10.1111/jam.15507

**Published:** 2022-03-08

**Authors:** Mirian F. Pimentel, Ali Y. Srour, Amanda J. Warner, Jason P. Bond, Carl A. Bradley, John Rupe, Martin I. Chilvers, J. Alejandro Rojas, Janette L. Jacobs, Christopher R. Little, Alison E. Robertson, Loren J. Giesler, Dean Malvick, Kiersten Wise, Albert Tenuta, Ahmad M. Fakhoury

**Affiliations:** ^1^ Department of Agricultural Sciences Southern Illinois University Carbondale Illinois USA; ^2^ USDA, ARS, New England Plant, Soil, and Water Laboratory Orono Maine USA; ^3^ Syngenta Crop Protection Raleigh‐Durham North Carolina USA; ^4^ Department of Plant Pathology University of Kentucky Research and Educational Center Princeton Kentucky USA; ^5^ Department of Entomology and Plant Pathology University of Arkansas Fayetteville Arkansas USA; ^6^ Department of Plant, Soil and Microbial Sciences Michigan State University East Lansing Michigan USA; ^7^ Department of Plant Pathology Kansas State University Manhattan Kansas USA; ^8^ Department of Plant Pathology and Microbiology Iowa State University Ames Iowa USA; ^9^ Department of Plant Pathology University of Nebraska‐Lincoln Lincoln Nebraska USA; ^10^ Department of Plant Pathology University of Minnesota Minneapolis Minnesota USA; ^11^ Department of Botany and Plant Pathology Purdue University West Lafayette Indiana USA; ^12^ Ontario Ministry of Agriculture Food and Rural Affairs (OMAFRA) Ridgetown Ontario USA

**Keywords:** environmental factors, *Fusarium* spp., *Glycine max*, seedling diseases, soilborne pathogens, *Trichoderma* spp.

## Abstract

**Aims:**

To isolate and characterize fungi associated with diseased soybean seedlings in Midwestern soybean production fields and to determine the influence of environmental and edaphic factors on their incidence.

**Methods and Results:**

Seedlings were collected from fields with seedling disease history in 2012 and 2013 for fungal isolation. Environmental and edaphic data associated with each field was collected. 3036 fungal isolates were obtained and assigned to 76 species. The most abundant genera recovered were *Fusarium* (73%) and *Trichoderma* (11.2%). Other genera included *Mortierella*, *Clonostachys*, *Rhizoctonia*, *Alternaria*, *Mucor*, *Phoma*, *Macrophomina* and *Phomopsis*. Most recovered species are known soybean pathogens. However, non‐pathogenic organisms were also isolated. Crop history, soil density, water source, precipitation and temperature were the main factors influencing the abundance of fungal species.

**Conclusion:**

Key fungal species associated with soybean seedling diseases occurring in several US production regions were characterized. This work also identified major environment and edaphic factors affecting the abundance and occurrence of these species.

**Significance and Impact of the Study:**

The identification and characterization of the main pathogens associated with seedling diseases across major soybean‐producing areas could help manage those pathogens, and devise more effective and sustainable practices to reduce the damage they cause.

## INTRODUCTION

Soybean (*Glycine max* [L.] Merr.) is an economically important crop worldwide and is considered to be essential for global food security (Hartman et al., 2011). Soybean production is dominated by Brazil, the United States and Argentina, which were responsible for 81% of total global production in the 2019/2020 growing season (USDA 2020). The United States is the world’s second largest soybean producer, with the majority of production concentrated in the Midwestern United States (USDA 2020). The impact of seedling diseases on soybean productivity is a major challenge to achieving maximum crop yield potential in soybean in all places it is grown. In fact, seedling diseases ranked third among diseases that consistently reduced soybean yields in the United States for the last 20 years (Bandara et al., 2020; Wrather et al., 2010). It is difficult to predict when seedling diseases will cause economic losses, due to environmental factors, variability in the pathogenicity of pathogen populations and pathogen interactions with the soil microbiome. Additionally, multiple pathogens often present in the same field require different management approaches, making seedling diseases difficult to manage and emphasizing the importance of accurate pathogen identification (Hartman et al., 2015).

Seedling diseases in soybeans are caused by a complex of pathogen species. The most commonly reported species include *Fusarium* spp., *Rhizoctonia solani*, *Phytophthora spp*. and *Pythium* spp. Soybean roots can be colonized by different fungal endophytes, including pathogenic and non‐pathogenic organisms (Fernandes et al., 2015; Impullitti & Malvick, 2013; Pimentel et al., 2006). Furthermore, multiple pathogenic species associated with seedling diseases can occur in the same field, which can hinder disease management (Díaz Arias, Leandro, et al., 2013; Díaz Arias, Munkvold, et al., 2013; Rojas et al., 2017a).

Several past studies aimed to identify pathogens associated with seedling diseases in soybean (Killebrew et al., 1993; Rizvi & Yang, 1996; Rojas et al., 2017a). In many instances, these studies focused on a limited geography or on a specific set of pathogens. In a study that was conducted in parallel to the research described in this paper, they concentrated on oomycete species (Rojas et al., 2017a, 2017b), whereas this study focused on fungal species. Rojas et al. (2017a, 2017b) identified oomycete species associated with soybean seedling diseases and documented the diversity and ecology of these communities. They identified a total of 84 oomycete species, 43 of which were confirmed to be pathogenic to soybean. The identified species belonged predominantly to the genus *Pythium* (94.85%), and remaining species included *Phytophthora*, *Phytopythium*, *Aphanomycces and Pythiogeton*. A total of 13 oomycete species characterized by Rojas et al. (2017a) had not been previously reported as root pathogens of soybean.

The abundance, diversity and pathogenicity can be influenced by edaphic and environmental factors (Rojas et al., 2017b; Srour et al., 2017; Yang & Feng, 2001). Soil temperature at planting, precipitation and soil type can influence pathogen development and exacerbate disease symptoms (Broders et al., 2007). Cultural practices that affect the composition of the soil microbial community can affect populations of soilborne pathogens and consequently the incidence of seedling diseases. Adequate planting depth, early planting, cropping history, cultivar selection, the adoption of cover crops and tillage, which can reduce the presence of primary inoculum in the vicinity of seedling roots, are practices that have been reported to potentially affect the incidence and severity of seedling diseases (Broders et al., 2007; Pankhurst et al., 1995).

Undesirable shifts in populations of soil microbes may result from edaphic modifications in adopted production systems that provide pathogen populations with competitive advantages, thus reducing the native disease suppressive capacity of the soil (Hartman et al., 2018; Srour et al., 2020; van Elsas et al., 2002). Therefore, the incidence and severity of diseases caused by soilborne pathogens are exacerbated by conditions favourable to pathogen development. Cool temperatures, compaction and wet soils can favour *Pythium* spp. and *Fusarium* spp. increasing the severity of root rot and or seedling damping off on soybean, whereas similar conditions with higher temperatures (>15°C) can favour *Phytophthora* spp. and *Rhizoctonia solani* causing root and stem rot (Winsor, 2020). Identifying environmental conditions that affect the incidence of specific pathogens in symptomatic soybean seedlings could ameliorate the current understanding of the aetiology of these diseases. This knowledge might have significant implications on the development and optimization of management strategies targeting seedling diseases. For instance, Rojas et al. (2017b) determined that latitude, longitude, precipitation, clay content and soil electrical conductivity were the most impactful factors that affected oomycete community composition in soybean fields with a history of seedling diseases.

The identification and characterization of the different fungi associated with seedling diseases in major soybean‐producing areas can provide valuable resources for research focused on seedling disease management, including the evaluation of fungicide resistance and development of effective and sustainable seed treatments, aiding breeding programmes set up priorities when targeting resistance to fungal pathogens, and testing and evaluating different management practices and their impact on seedling diseases. In this study, we characterized the culturable fungal community associated with soybean seedlings across eight large soybean‐producing states in the Midwestern United States. The objectives of the study were to: (i) identify the fungal species associated with soybean seedlings across major soybean‐producing states in the United States; and (ii) determine the influence of several environmental and edaphic factors on the occurrence and abundance of these fungal species.

## MATERIALS AND METHODS

### Sample collection and fungal isolation

**FIGURE 1:**
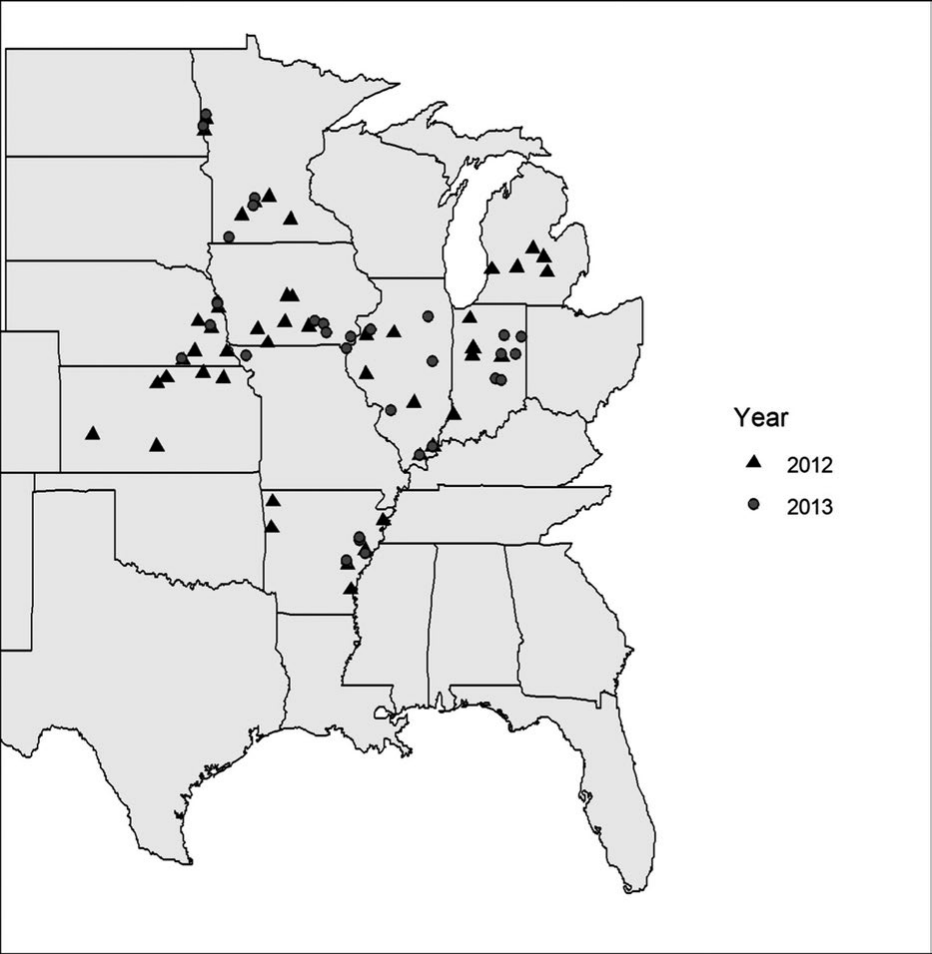
Midwest USA map of the sampled soybean fields in 2012 and 2013

A survey was conducted across eight US states—Arkansas, Illinois, Indiana, Iowa, Kansas, Michigan, Minnesota and Nebraska—during 2012 and 2013 (Figure 1). Between five and eight fields were sampled per participating state, with a total of 49 fields sampled in 2012 and 47 fields sampled in 2013. Fields with history of seedling disease or plant stand issues were selected. Collaborators from each state collected 25 soybean seedlings from each of the fields in that state following a standard sampling procedure described by Rojas et al. (2017a). Twenty‐five seedlings with above‐ground symptoms were collected from a W‐shaped transect across each field. In fields where not enough symptomatic seedlings were found, seedlings were randomly sampled. Due to crop rotation practices, the fields sampled in 2012 were different from the fields sampled in 2013. The growth stage at which seedlings were sampled varied from VE to V4. Seedlings were transported in coolers with ice and processed within 24 h after collection by the collaborators in each state following a standard protocol as follows: seedlings were prepared for isolation by washing their roots under running tap water until all visible soil was removed. Seedling roots were then disinfected by soaking in a 1% NaOCl solution for 30 s, followed by a thorough rinse in distilled water for 1 min. Seedlings were then dried with a sterile paper towel to remove excess water. Root sections (0.5–1 cm) were cut from diseased tissue, including the edge of the disease lesions, using a sterile scalpel. The root sections were placed onto water agar media plates amended with streptomycin (30 mg/L). Ko and Hora media (Ko & Hora, 1971) was used in 2013, in addition to water agar media, to increase the recovery of *R. solani*. The plates were incubated in the dark at 20–22°C for 7 days and were checked daily for hyphal growth. Hyphal tips that were observed growing were transferred to new potato dextrose agar (PDA) amended with ampicillin (50 mg/L) and tetracycline (50 mg/L). Pure colonies were labelled and stored as plates at 4**°**C until transferred to new PDA plates for molecular identification.

The objective of this research was to focus on fungal isolates, while the oomycete isolates were identified and characterized by Rojas et al. (2017a, 2017b). Collaborators shipped all fungal isolates to Southern Illinois University Carbondale (SIUC).

### Isolate identification and fungal storage

Fungal isolates were identified using PCR and subsequent sequencing of the internal transcribed spacer (ITS) of nuclear ribosomal RNA, using the primer pairs ITS1 and ITS4 (White et al., 1990). Speciation within the *Fusarium* genus was based on the translation elongation factor (EF1‐*α*) gene using a nested PCR with the primers EF1 and EF2 (O’Donnell et al., 1998) and Alfie1 and Alfie2 (Yergeau et al., 2005). To confirm the identity of ambiguous isolates, the intergenic spacer (IGS) region of the ribosomal RNA was also sequenced using the primers LR12R and invSR1R (Vilgalys et al., 1994). As per Alshahni et al. (2009), total of 70 μl of alkaline lysis buffer (ALB; 20 mM Tris HCl pH 8.0, 5 mM EDTA, 400 mM NaCl, 0.3% SDS, 200 μg/ml proteinase K) was added to a 2‐ml Eppendorf tube. A pin head‐sized piece of mycelia was collected from 7‐ to 10‐day‐old cultures using a sterile toothpick. Mycelia were added to the ALB buffer and incubated at 55°C for 2 h, 95°C for 10–15 min, placed on ice for 3 min and centrifuged for 5 min at 10,600 *g*. PCR mixes consisted of 1 U Taq DNA Polymerase (GenScript), 1× reaction Buffer (containing 2 mM Mg^2+^), 0.2 mM dNTP mix, 0.25 μM of each primer, 0.6 μg/ml of bovine serum albumin, 0.5–2 μl of fungal DNA and sterile distilled water to a final 50 μl reaction volume. The amplification program for the reactions targeting the ITS region consisted of 95°C for 2 min initial denaturation; 35 cycles of 95°C for 30 s denaturation; 50°C for 1 min annealing; 72°C for 1 min elongation; and 72°C for 10 min final extension. The amplification program for the reactions targeting the EF1‐α region consisted of 94°C for 3 min initial denaturation; 35 cycles of 94°C for 30 s denaturation; 52°C for 30 s annealing; 72°C for 1 min elongation; and 72°C for 10 min final extension. The amplification program for the reactions targeting the IGS region consisted of 94°C for 3 min initial denaturation; 35 cycles of 94°C for 30 s denaturation; 55°C for 1 min annealing; 72°C for 2 min and 30 s elongation; and 72°C for 10 min final extension. Amplicons were purified by adding 5 μl of a mixture of 3 U of exonuclease I (Thermo Scientific) and 0.5 U of shrimp alkaline phosphatase (Affymetrix) with 30 min incubation at 37°C, followed by 85°C for 15 min to deactivate the enzymes. Amplicons were sequenced by Sanger sequencing at SIUC. The Four Peaks software (www.nucleobytes.com) was implemented to visualize the sequences and to crop unnecessary noise on the 3′ and 5′ ends. Sequences were deposited in GenBank under accession numbers MK593627–MK595448 and MN451718–MN452860 for ITS, MN553711–MN555299 for EF, and MN555300–MN555327 for IGS.

To identify the fungal isolates, the ITS sequences were primarily queried against the NCBI fungal database using a BLASTn search algorithm (Altschul et al., 1990). The isolates were assigned to distinct species using blastn with an e‐score cutoff of <10^−4^ and minimum 97% percent similarity. To identify species within the *Fusarium* genus, EF sequences were queried against the latest versions of two curated databases, Fusarium MLST (http://www.cbs.knaw.nl/Fusarium) and Fusarium‐ID (http://isolate.fusariumdb.org).

All identified fungal isolates were used to build a fungal collection for long‐term storage using a filter paper method described by Fong et al. (2000). The fungal isolates were grown for 5–7 days over sterile Grade 3 Whatman filter paper pieces placed on PDA. Full strength PDA was used, and the plates were incubated at 25°C in the dark until sporulation. The filter paper pieces covered with spores were air dried for 8 h in a laminar flow hood, placed into sterile labelled glassine envelopes and stored at −20°C.

### Environmental and edaphic variables

Edaphic and environmental parameters prevailing in the sampled fields were collected as described by Rojas et al. (2017b). Information about environmental and edaphic (soil) factors associated with each sampled field was obtained using GIS coordinates to retrieve data from publicly available databases. The variables of interest were precipitation (millimetres), temperature (°C), previous crop, slope (^0^), available water capacity (cm water/cm soil), cation exchange capacity (milliequivalents/100 g of soil at pH 7.0), clay content (%), sand content (%), silt content (%), soil organic matter (%), soil bulk density 1/3 bar (gr/cm^3^), water content 1/3 bar (volumetric percentage of the whole soil), surface texture, soil pH and water source (irrigated or rain‐fed). Data pertaining to the soil physical and chemical characteristics were retrieved from the Natural Resources Conservation Service soil database (https://www.nrcs.usda.gov/). The yearly temperature and precipitation data were retrieved from the PRISM Climate Group (http://www.prism.oregonstate.edu/). Information about topology was obtained from the United States Geological Survey (https://www.usgs.gov/), while data related to land usage were retrieved from the USDA National Agricultural Statistics Service (https://nassgeodata.gmu.edu/ CropScape/).

### Statistical analysis

A fungal species table was created based on the molecular identification of the isolates recovered from the collected soybean seedlings. Species with an abundance and frequency <10% were excluded from further analysis. The diversity within each field (alpha diversity) was estimated using the Shannon‐Wienner Index, Simpson Index, Pilou’s Evenness and richness through the vegan package (Oksanen, 2015) in R. In order to study the fungal diversity between communities (beta diversity), the Bray–Curtis dissimilarity index (Bray & Curtis, 1957) was calculated based on the species abundance. The resulting dissimilarity matrices were used to perform a Permanova analysis for the categorical variables ‘state’, ‘year’, ‘water source’, ‘previous crop’ and ‘surface texture’. The Permanova analysis was followed by a pairwise Adonis test (Martinez, 2019) to test the statistical significance of all pairs of samples with regard to ‘previous crops’, ‘water source’ and ‘surface texture’, with 9999 permutations. To investigate the effect of the main environmental factors on beta diversity, a canonical correspondence analysis (CCA) (Økland & Eilertsen, 1994) was performed using the species composition data over the study sites as a function of different environmental variables. A Kendall’s correlation test was performed to determine the effect of each environmental and edaphic parameters on each fungal species (Kendall & Gibbons, 1990). Fields were kept separate and were not grouped by state given the variation in environmental and edaphic characteristics across these geographic locations, even within a state. A total of 12 environmental and edaphic factors were tested using the Kendall’s correlation against the top abundant fungal species.

## RESULTS

### Identification and diversity of isolated fungi

**FIGURE 2:**
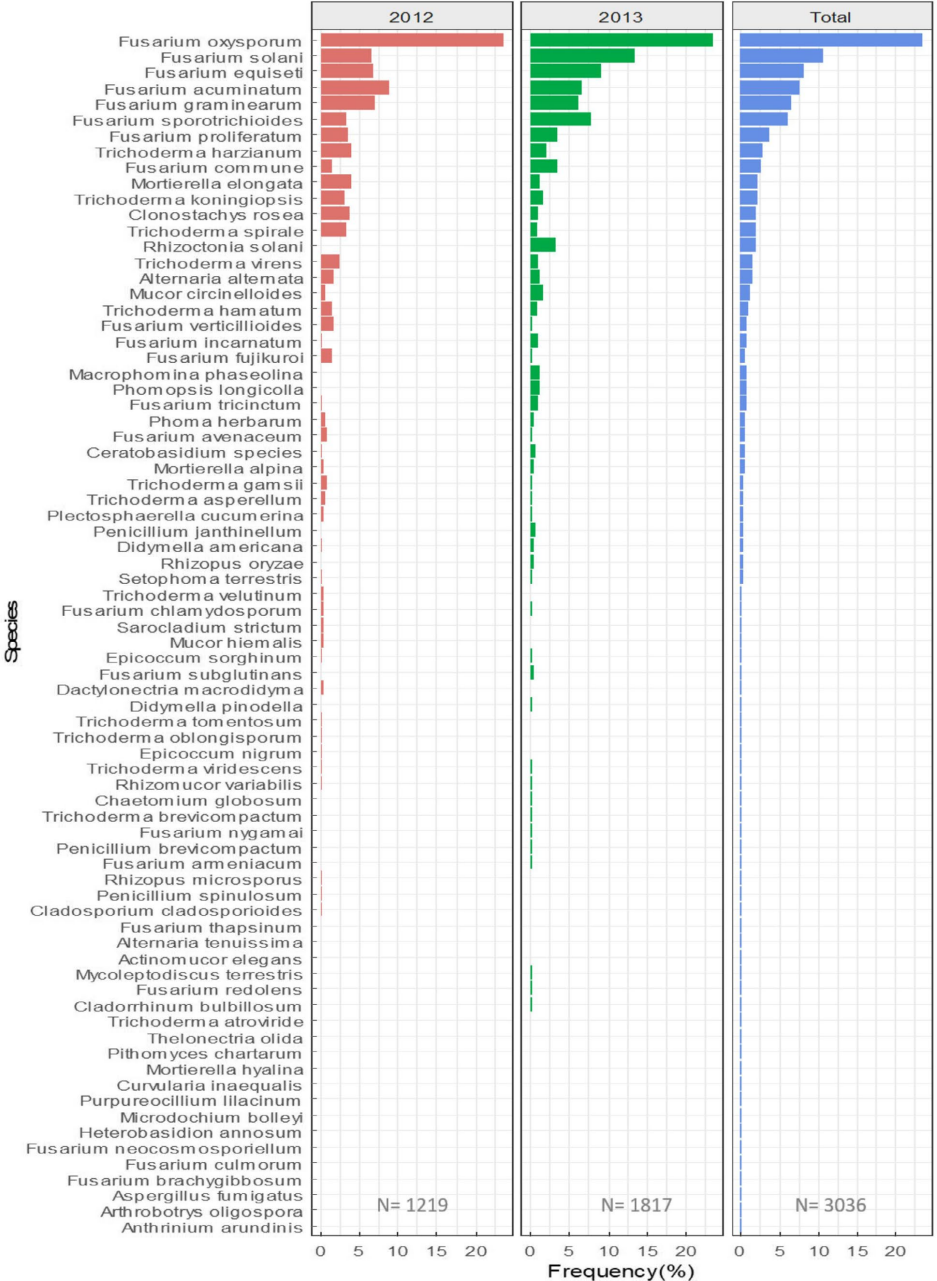
Frequency of isolation of fungal species from soybean roots in 2012, 2013

During both years of this study, 96 fields were sampled across 8 states, 49 fields sampled in 2012 and 47 fields samples in 2013 (Figure 1). A total of 3036 fungal isolates were recovered from soybean seedlings' roots: 1219 recovered in 2012 and 1817 recovered in 2013. The 3036 isolates were assigned to 76 species. A complete list of the identified species is available in File S1. The most abundant genera recovered in both years were *Fusarium* (73.0%), *Trichoderma* (11.2%), *Mortierella* (2.8%), *Clonostachys* (2.1%), *Rhizoctonia* (1.9%), *Alternaria* (1.5%), *Mucor* (1.4%), *Macrophomina* (0.8%), *Phomopsis* (0.7%), *Penicillium* (0.5%), *Phoma* (0.5%), *Ceratobasidium* (0.5%), *Didymella* (0.5%), *Epicoccum* (0.4%), *Rhizopus* (0.4%), *Plectosphaerella* (0.3%), *Setophoma* (0.3%) and *Sarocladium* (0.2%). The remaining identified genera had a relative abundance of less than 0.2% individually (Figure 2).

**FIGURE 3:**
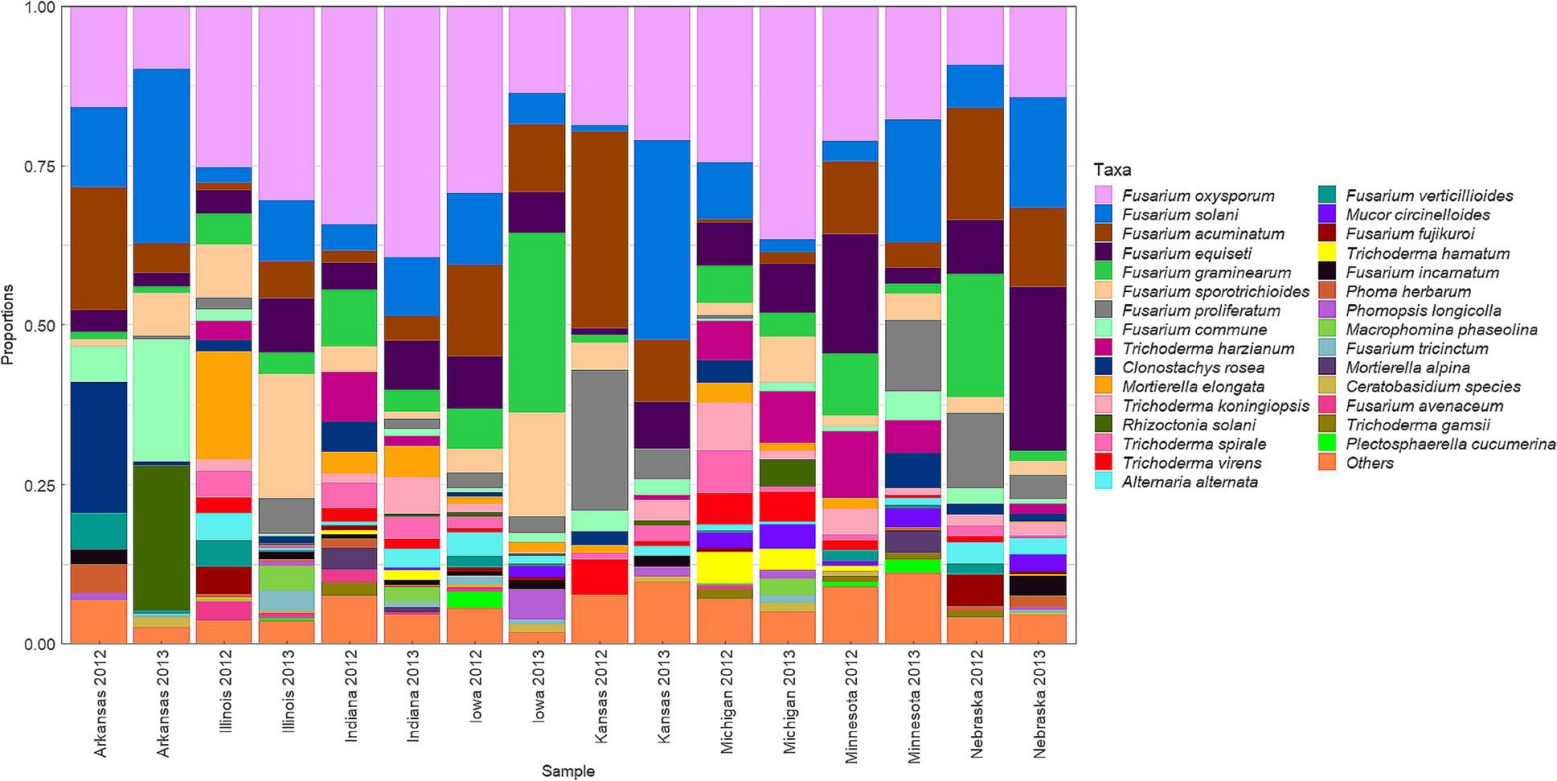
Abundance of the top 35 fungal species recovered from diseased soybean across locations in 2012 and 2013

*Fusarium* was the most abundant genus in all fields, constituting 66.6% and 77.3% of total species recovered in 2012 and 2013, respectively (Figure 2). The profiles of isolation for the *Fusarium* species were similar in both years across all states (Figure 3). A total of 22 *Fusarium* species were identified, with *F. oxysporum* (23.5%), *F. solani* (10.7%), *F. equiseti* (8.2%), *F. acuminatum* (7.6%), *F. graminearum* (6.6%), *F. sporotrichioides* (6.1%), *F. proliferatum* (3.6%) and *F. commune* (2.6%) being the most frequently isolated species. The other *Fusarium* species recovered were *F. verticillioides*, *F. avenaceum*, *F. incarnatum*, *F. fujikuroi*, *F. tricinctum*, *F. subglutinans*, *F. thapsinum*, *F. redolens*, *F. culmorum*, *F. chlamydosporum*, *F. armeniacum*, *F. brachygibbosum*, *F. neocosmosporiellum* and *F. nygamai*, all with an individual abundance lower than 1% (Figure 2).

The second most abundantly recovered genus, *Trichoderma*, composed 17.6% of the total isolates in 2012 and 6.9% in 2013. The isolated *Trichoderma* spp. included *T. harzianum* (2.9%), *T. koningiopsis* (2.2%) *T. spirale* (1.9%), *T. virens* (1.6%), and *T. hamatum* (1.0%) (Figure 2). *Trichoderma* species recovered with relative abundance lower than 0.5% included *T. asperellum*, *T. gamsii*, *T. velutinum*, *T. viridescens*, *T. brevicompactum*, *T. tomentosum*, *T. oblongisporum* and *T. atroviridae* (Figure 2). Other fungal species isolated in this study were *Rhizoctonia solani* (1.9%), *Alternaria alternata* (1.5%*)*, *M. phaseolina* (0.8%) and *Clonostachys rosea* (2.1%).

### Species diversity per field across states

**FIGURE 4:**
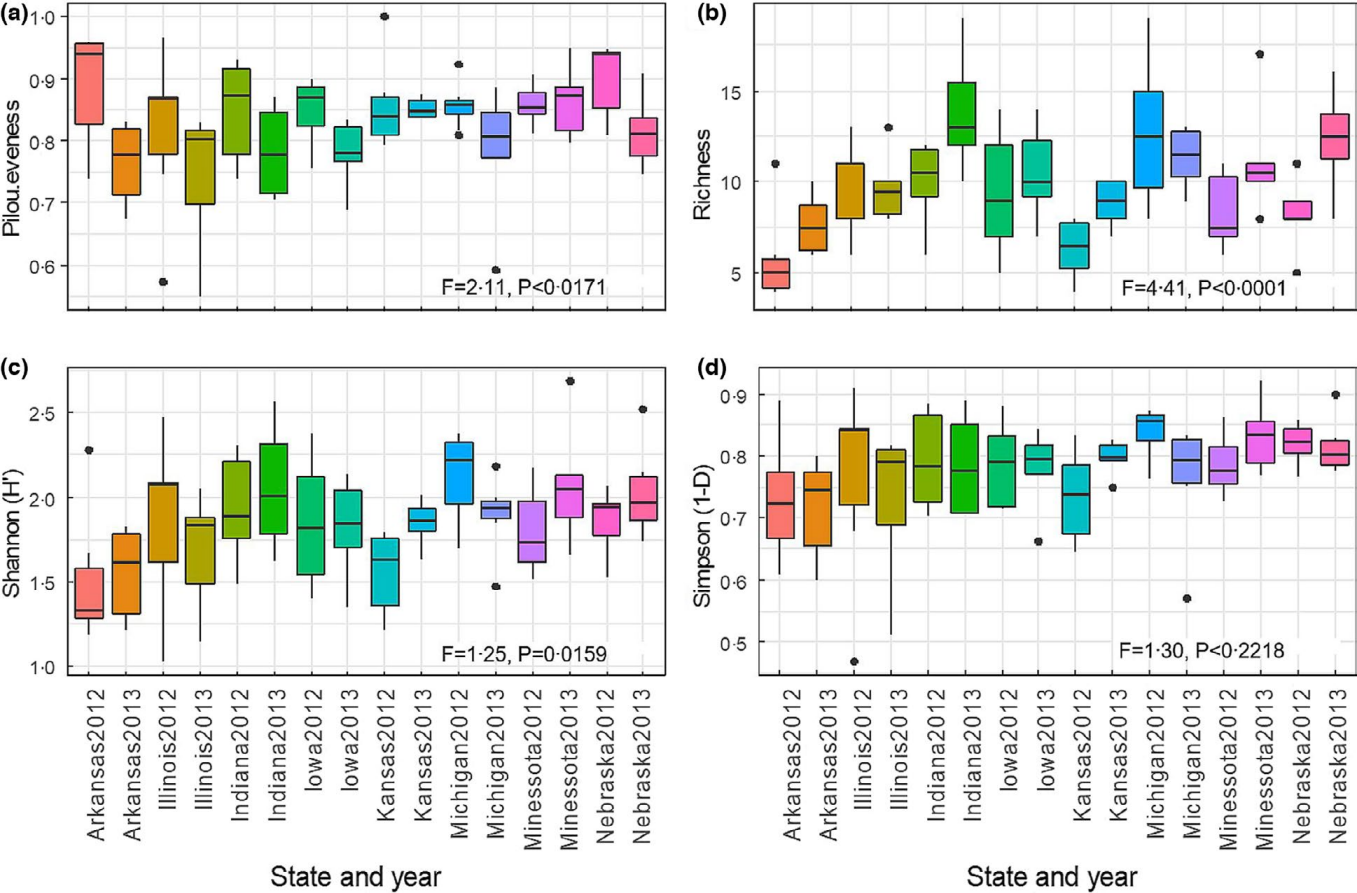
Means (±SE) of Pilou evenness (a), species richness (b), and alpha diversity indices (c, d) calculated for the fungal isolates recovered within fields for each state in 2012 and 2013. Richness is the number of species in each sampled field (averaged by state in each year). Evenness was measured using Pilou’s evenness. The alpha diversity within the surveyed fields was assessed by calculating the Shannon‐Wiener index and the Simpson index using the vegan package in R

The diversity within the surveyed fields (alpha diversity) was assessed by calculating the Shannon‐Wiener index, the Simpson index and the Pilou’s Evenness (Pielou, 1966). The average number of species per field across different states ranged from 5.8 to 13.8 (Figure 4; Table S1). Indiana 2013 and Michigan 2012 had the highest species richness, with an average count of 13.8 and 12.6 observed species per field, respectively. Arkansas in 2012 and Kansas in 2012 had the lowest richness, with 5.8 and 6.3 average number of species, respectively (Figure 4; Table S1). The average Shannon–Wiener index (H′) per state and year ranged from 1.51 to 2.13, which reflects a low to moderate diversity (Figure 4). The calculated Shannon–Wiener index had the highest values for Michigan 2012, Indiana 2013, Minnesota 2013 and Nebraska 2013, with average H′ of 2.13, 2.05, 2.07 and 2.03, respectively. Lower H′ values were noted in Arkansas 2012 (1.51), Arkansas 2013 (1.55) and Kansas 2012 (1.55) (Figure 4). The Simpson’s index of diversity ranges from 0 to 1, with higher values representing higher diversity. The average Simpson’s index of diversity in the targeted fields ranged from 0.72 to 0.84, indicative of a moderate diversity (Figure 4). However, no statistically significant differences were noted for the Simpson’s index of diversity across different states and years (Figure 4; Table S1). Differences in species evenness were found across fields from different states and years. Illinois 2013 and Arkansas 2013 showed intermediate evenness with values close to 0.75, whereas Nebraska 2012 and Arkansas 2012 showed the highest evenness, 0.90 and 0.88, respectively (Figure 4; Table S1). Intermediate evenness values were evidenced by relatively higher abundance of two species in comparison to the others, such as *F. sporotrichioides* and *F. oxysporum* in Illinois 2013 and *R. solani* and *F. solani* in Arkansas 2013 (Figure 3).

### Influence of abiotic factors on the community structure

**FIGURE 5:**
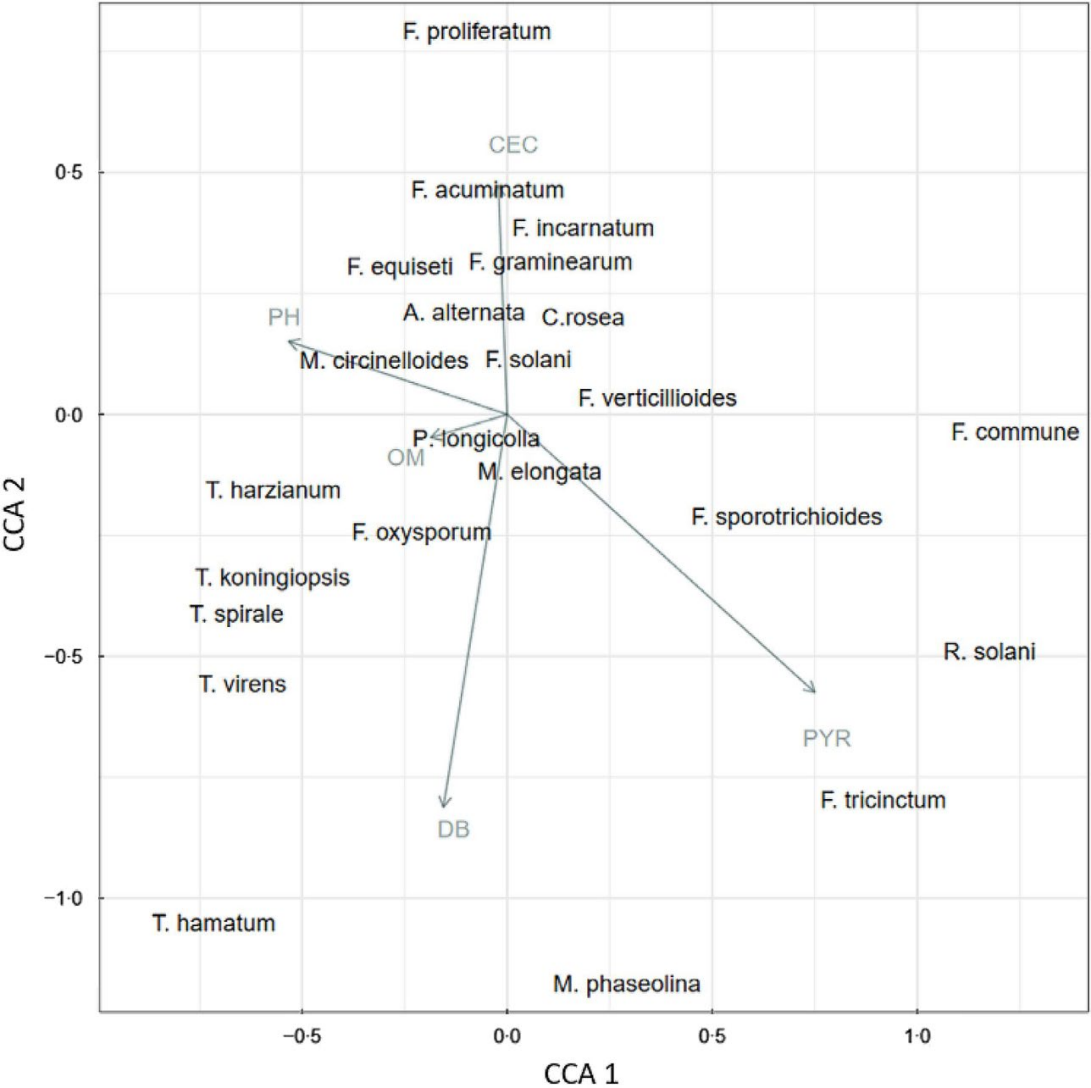
Canonical correspondence analysis (CCA) scaling type 2 plot of the fungal community structure isolated from diseased soybean seedlings in the Midwest USA. Environmental variables that significantly influence the community structure are plotted as vectors based on correlations with species composition. CEC: Cation exchange capacity (milliequivalents/100 g of soil at pH 7.0), DB: soil bulk density 1/3 bar (g/cm^3^), OM: soil organic matter (%), PH: soil pH, PYR: precipitation year (ml)

Yearly average precipitation, soil bulk density 1/3 bar, cation exchange capacity, soil pH and organic matter appeared to be the major abiotic factors associated with the fungal community structure (*p* = 0.05) (Figure 5).

**FIGURE 6:**
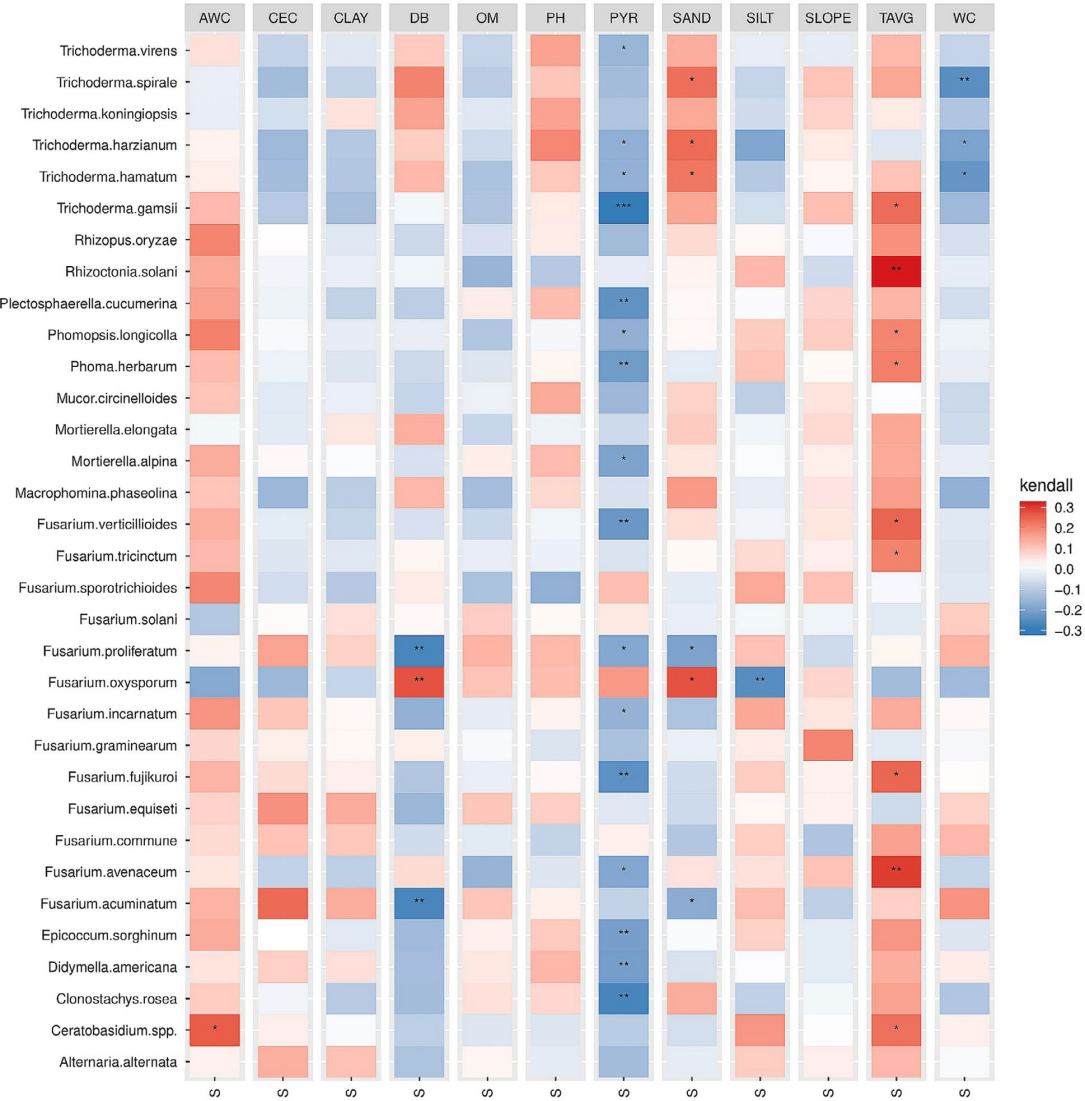
Heatmap showing correlations between environmental and soil edaphic factors with the top abundant fungal species isolated from diseased seedlings. Colours represent Kendall’s correlation coefficients (τ) (Kendall & Gibbons, 1990) between relative abundances of the top fungal species and environmental parameters. Asterisks (*) indicate the significance level for Kendall’s rank correlation (*p <*0.05*, 0.01**, 0.001***). AWC: available water capacity (cm water/cm soil), CEC: cation exchange capacity (milliequivalents/100 g of soil at pH 7.0), CLAY: clay content (%), DB: soil bulk density 1/3 bar (g/cm^3^), OM: soil organic matter (%), PH: soil pH, PYR: precipitation year (ml), SAND: sand content (%), SILT: silt content (%), SLOPE: slope (°C), TAVG: temperature year (°C), WC: water content 1/3 bar (volumetric percentage of the whole soil)

**FIGURE 7:**
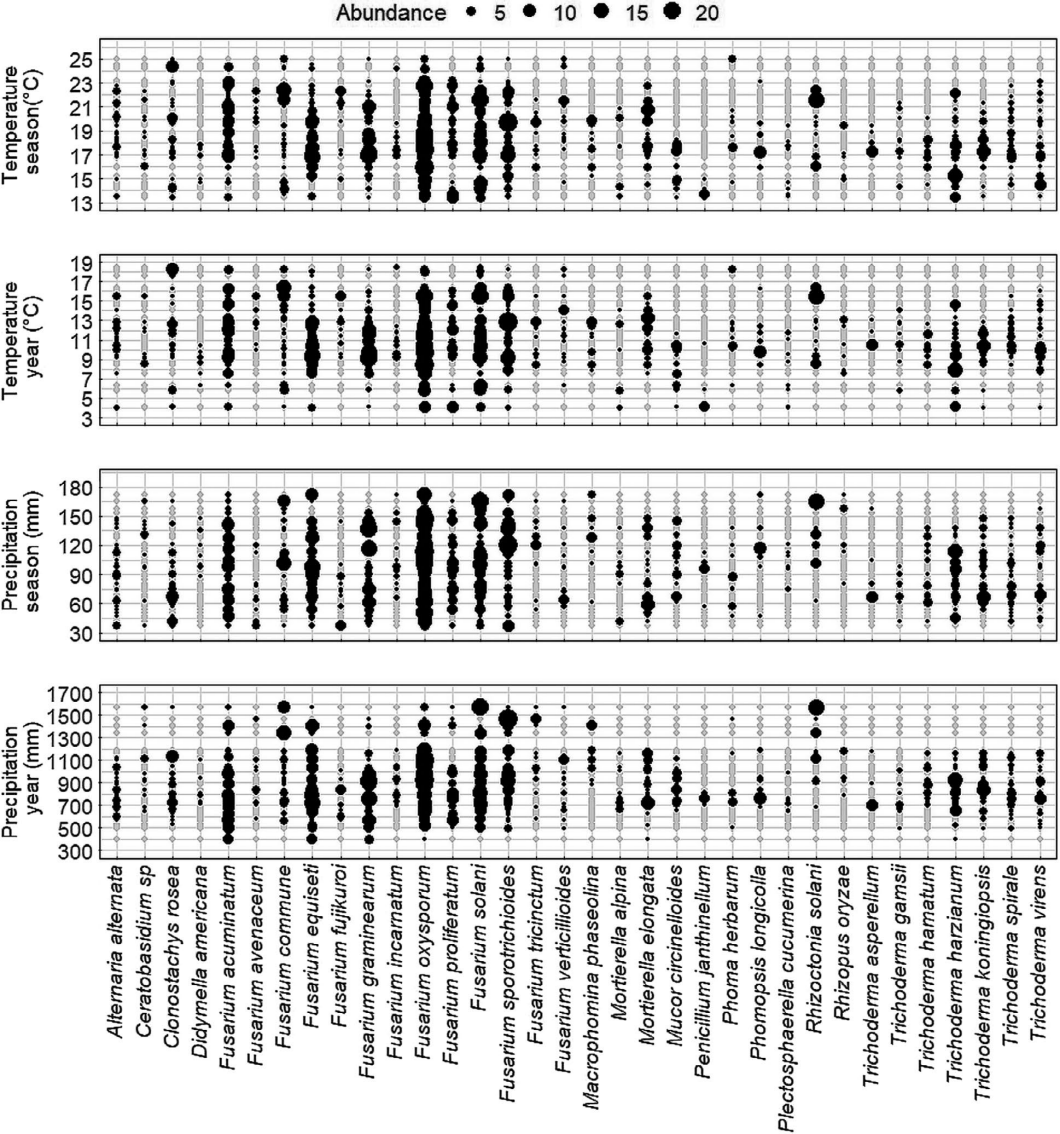
Distribution of the abundance of the top isolated fungal species across the temperature and precipitation gradients measured at the beginning of the growing season (April to July) and as a year average

In total, 32 fungal species profiles were obtained from 96 field samples collected in 2012 and 2013, which were used to determine correlations with 12 environmental variables (File S1). A heatmap of the correlations between the environmental variables and filtered fungal species associated with diseased seedlings is depicted in Figure 6. Among the investigated environmental variables, yearly average precipitation and yearly average temperature showed the strongest correlations with fungal species. Most isolated fungal species apart from *Fusarium* spp. were negatively correlated with yearly average precipitation (τ = −0.1 to −0.3) (Figure 6). Conversely, the incidence of most fungal species correlated positively with average temperature (τ =0.1 to 0.3). Among the edaphic factors, sand content showed positive correlation with *Trichoderma* species (τ =0.2) and *Fusarium oxysporum* (τ =0.3), and negative correlation with *F. proliferatum* (τ = −0.2) and *F. acuminatum* (τ = −0.2). Water content was negatively correlated with *T. spirale*, *T. harzianum* and *T. hamatum* (τ = −0.2). Soil bulk density was positively correlated with *F. oxysporum* (τ =0.3), whereas *F. proliferatum* and *F. acuminatum* were negatively correlated (τ = −0.3) with soil bulk density. In general, we noted opposite trends with *F. oxysporum* in comparison to the other *Fusarium* species. The correlations with available water capacity, cation exchange capacity, clay content, organic matter, water pH, silt content and slope with individual species were not significant (Figure 6). The distribution of the abundance of the top isolated fungal species across the temperature and precipitation gradients are displayed in Figure 7.

There were significant differences between different states in the incidence of fungal species isolated from soybean seedlings (*p* =0.0001; Table 1). The effect of the ‘year factor’ on the incidence and abundance of fungal isolates was also significant (*p* =0.0001, Table 1). ‘Water source’, ‘previous crop’ and ‘surface texture’ also influenced the community structure of fungal isolates associated with diseases based on the Permanova tests (*p* <0.05, Table 1). Different fungal communities were noted following soybean, corn and grassland/pasture, suggesting an effect of previous crops on the fungal community. Soil type and texture (amount of clay, sand and/or silt) also seemed to have influenced the community. Fungal communities in silty clay and silty loam soils (sand content <40%) were significantly different from those in loam soils (sand content >40%) as shown in (Table 1). Moreover, water source exhibited a significant effect on fungal diversity (*p <* 0*.05*), with rain fed soybean plots harbouring fungal communities distinct from those identified in irrigation supplied fields (Table 1).

**TABLE 1:** Permanova analysis of categorical variables influencing fungal community structure (beta diversity) associated with soybean seedlings across different states on Bray–Curtis distances

	Df	SS	MS	Pseudo‐F^a^	*p* (perm)^a^
Main effects					
State	7	43,677	6239.6	5.2982	0.0001***
Year	1	8717.4	8717.4	7.4021	0.0001***
Previous crop	4	14,549	3637.2	1.8339	0.0037**
Surface texture	8	20,859	2607.4	1.3147	0.0517*
Water source	1	4270	4270	2.1529	0.0128*
*Interaction effects*					
State × Year	7	24,936	3562.2	3.0248	0.0001***
P. crop × S. texture	10	22,364	2236.4	1.1276	0.2260
P. crop × W. source	1	2551.2	2551.2	1.2863	0.2093
S. texture × W. source	2	5569.5	2784.7	1.4041	0.1251
Residuals	67	1.3E+05	1983.3		
Total	93	2.0E+05			
Pairwise comparison	*t*	*p*(perm)^a^			
Soybean versus Corn	1.7985	0.0006***			
Soybean versus Grassland/Pasture	1.3293	0.0557*			
Corn versus Grassland/Pasture	1.4891	0.0112*			
Silt loam, loam	1.5507	0.0044**			
Silty clay, loam	1.7323	0.0002***			
Silty clay, clay loam	1.4639	0.0931			

Abbreviations: df, degrees of freedom; MS, mean sum of squares; SS, sum of squares.

^a^ Pseudo‐*F* values and *p* values based on 9999 permutations.

Significance levels *p <0.05, **p <0.01, ***p <0.001.

## DISCUSSION

Seedling diseases and root rot pathogens of soybean reduce yields in the major US soybean‐producing states significantly. Their diagnosis and management can be challenging, and it is often difficult to predict when seedling diseases will be severe in a specific location and year given the complexity of factors affecting their incidence and severity. Characterization of predominant pathogens associated with seedling diseases across major soybean‐producing areas could improve management efforts, ultimately leading to more effective and sustainable practices to mitigate impacts of seedling disease.

In this study, we identified 76 fungal species associated with soybean seedlings collected from fields where seedling diseases have been problematic. Although no pathogenicity tests were conducted in this study to determine the pathogenicity of the collected isolates on soybean, several of the isolates belonged to species previously documented to be soybean pathogens. Regardless of location, the majority of fungal isolates recovered in this study were of the order Hypocreales, with *Fusarium* (71%) being the most abundant genus. Of the 17 *Fusarium* species isolated, *F. oxysporum*, *F. solani*, *F. equiseti*, *F. acuminatum*, *F. graminearum*, *F. sporotrichioides* and *F. proliferatum* were the most abundantly recovered. Similarly, a 3‐year survey conducted in Iowa identified 15 *Fusarium* spp. associated with soybean roots, with *F. oxysporum*, *F. acuminatum*, *F. graminearum* and *F. solani* as the most frequent and widespread species (Díaz Arias, Leandro, et al., 2013; Díaz Arias, Munkvold, et al., 2013). Several of the *Fusarium* species identified in the present study are reported to be pathogenic to soybean. For instance, *F. solani*, *F. oxysporum*, *F. proliferatum*, *F. graminearum* and *F. sporotrichioides* are known causal agents of soybean root rot (Abdelmagid et al., 2020; Broders et al., 2007; Chang et al., 2015; Díaz Arias, Leandro, et al., 2013; Díaz Arias, Munkvold, et al., 2013; Farias & Griffin, 1989; Killebrew et al., 1993; Pioli et al., 2004; Rizvi & Yang, 1996). *Fusarium redolens* has been reported to cause root rot in Minnesota soybean fields (Bienapfl et al. 2010). *Fusarium fujikuroi* has also been reported to cause pre‐ and post‐emergence damping‐off on soybean (Chang et al., 2020; Pedrozo et al., 2015). *Fusarium thapsinum* and *F. equiseti* have also been reported to be seedborne pathogens of soybean (Pedrozo & Little, 2014). *Phomopsis longicolla* and *Alternaria alternata* that were also recovered in this study are known seedborne pathogens of soybean (Kunwar et al., 1986; Li et al., 2010) It is to be noted that, in this study, the seeds were not tested prior to planting to ensure that they pathogen free. Therefore, it is possible that some of the pathogens that we isolated originated from contaminated seeds. Other well‐known soybean pathogens isolated in this study were *R. solani*, the causal agent of Rhizoctonia damping‐off and root rot of soybean (Ajayi‐Oyetunde & Bradley, 2017) and *M. phaseolina*, the causal agent of charcoal rot (Romero Luna et al., 2017).

In this study, *T. harzianum*, *T. spirale*, *T. koningiopsis*, *T. virens* and *T. hamatum* were isolated at relatively high frequency from diseased roots. These isolated *Trichoderma* spp. have been reported in the literature to mycoparasitize and antagonize plant pathogens such as *Fusarium* spp., *R. solani*, *A. alternata* and *M. phaseolina* (Harman, 2000; Howell, 2003; Mukherjee et al., 2012; Verma et al., 2007). In a separate study evaluating the diversity of endophytic fungi in soybean, *Trichoderma* was the second most abundant genus (16.9%) after *Fusarium* (39.7%), to be isolated from soybean roots (Yang et al., 2018) which is consistent with our findings. Conversely, other studies did not report *Trichoderma* spp. as part of the soybean fungal community (Fernandes et al., 2015; Dean et al., 2016; Pimentel et al., 2006) or reported substantially lower frequency—<4%—of isolation of *Trichoderma* spp. (Impullitti & Malvick, 2013) from soybean roots. In this study, the higher abundance of *Trichoderma* spp. recovered in 2012 (17.6%) versus 2013 (6.9%) might have been due to the lower precipitation and warmer temperatures at the beginning of the growing season in 2012 compared to 2013, which might have favoured *Trichoderma* spp. over other fungal species. In addition, differences in culture media used in both years possibility affected the recovery rate of isolates. The high frequency of recovery of *Trichoderma* spp. may be attributed to the saprophytic habits of those species. It should be noted that all *Trichoderma* species isolated in this study are considered to be endophytes (Contreras‐Cornejo et al., 2016; Druzhinina et al., 2011). They have been reported to associate with the roots of host plants and to perform critical ecological functions, including disease suppression, improving nutrient solubilization and uptake, stimulation of plant growth and health, and reduction of abiotic stresses (Bucio et al., 2015; Harman, 2006; Shi et al., 2012; Yedidia et al., 1999). Nevertheless, the beneficial attributes of these associations to soybean plants may also depend on other factors such as the abundance of these beneficial species in the soil and other abiotic and biotic factors that affect their activity (Burpee, 1990). It is to be noted here that several *Trichoderma* isolates from this study have been tested in related work and were shown to demonstrate a strong antagonistic activity against *F. virguliforme*. In fact, some of the tested isolates significantly reduced sudden death syndrome (SDS) foliar symptoms and root rot on soybean caused by *F. virguliforme* in both greenhouse and microplot experiments (Pimentel et al., 2020).

*Clonostachys rosea—*constituting 2% of the fungal isolates recovered in this study–has been reported to be a biological control agent with activity against important phytopathogens, including *Sclerotinia sclerotiorum*, *F. graminearum*, *and R. solani* (Gimeno et al., 2019; Wu et al., 2018; Karlsson et al., 2015; Salamone et al., 2018). However, *C. rosea* was also reported to be a potential pathogen, capable of causing root rot, interveinal chlorosis and marginal necrosis on soybean seedlings (Bienapfl et al., 2012).

Our results also suggest that the incidence and severity of soybean seedling diseases and the composition of pathogen populations that cause them are dependent on abiotic factors and location. This is in accordance with other studies indicating that microbial community patterns in the soil are primarily related to spatial, biotic, and abiotic factors (Srour et al., 2017; Yergeau et al., 2010). Similarly, studies have shown that several biotic factors, such as the susceptibility of the plant host and the interaction with other microbes present in the soil are key factors that shape the composition of the fungal community.

The previous crop used in a cropping system impacts the composition of the microbial community in a given location by favouring reproduction of either pathogenic and/or mutualistic organisms that are closely associated with that plant host (Benitez et al., 2017; Edwards et al., 2015). Our results suggest that the *Fusarium* community structure was strongly influenced by the ‘previous crop’ factor. *Fusarium graminearum*, *F. oxysporum*, *F. proliferatum*, *F. acuminatum*, and *F. equiseti*, were prevalent when corn was the previous crop, whereas *F. solani* had higher abundance under continuous soybean. Interestingly, *T. harzianum*, *T. koningiopsis*, *C. rosea* and *R. solani* were also more abundant under continuous soybean, whereas *T. hamatum*, *T. spirale*, *T. virens* and *M. elongata* were more abundant in soybean after corn. Although not tested in this study, the pathogen populations and the overall plant host associated microbial community can be affected by other crucial factors such as: cover cropping, host genotype, plant growth stage, seed treatment, fertigation and tillage practices (Acharya et al., 2019; Longley et al., 2020).

In this study, the abundance of specific pathogens associated with seedling diseases was significantly affected by environmental and soil edaphic factors, previous crop, year and by field location. The complexity of fungal communities in soil ecosystems is related to compositional changes caused by differences in edaphic and environmental variables (Dean et al., 2016; Jonhman et al., 1995). Rojas et al. (2017b) studied the oomycete community structure associated with soybean seedling diseases in a similar study. Their results also indicate seasonal temperature, seasonal precipitation, clay content, latitude and longitude as factors explaining the variability observed in the community composition. Environmental factors and conditions are especially influential on seedling diseases which are more likely to occur under cool, wet conditions at the early stages of plant development (Arias et al. 2013). The average yearly temperature across the various locations ranged from a minimum of 4°C to a maximum of 19°C. Our results highlighted a positive correlation between the abundance of several fungal species and the average yearly temperature, including *R. solani* which is known to be favoured by warmer temperatures (>15°C) (Dorrance et al., 2003). Due to shifts in the global climate patterns influenced by climate change, temperature and precipitation are expected to increase. In the United States, many regions are already experiencing dramatic changes in weather patterns which can alter the traditional range of some pathogens, and thus affect the disease pressure they cause on crops (Delgado‐Baquerizo et al., 2020; Velásquez et al., 2018). In fact, increasing temperatures may favour pathogens such as *M. phaseolina* and *C. sojina*, which may consequently be capable of surviving winters in more Northern growing regions. Conversely, increasing temperatures might reduce the abundance and the impact of other pathogens such as *Sclerotinia sclerotiorum* in more northerly surveyed areas (Velásquez et al., 2018).

In this work, key fungal species associated with soybean seedling diseases occurring in several US production regions were characterized. This work also identified major environment and edaphic factors that affected the abundance and occurrence of these species. Not surprisingly, most of the recovered species were pathogens known to cause root rot, seedling decay and damping‐off of soybean. However, non‐pathogenic organisms, including putative biocontrol agents, were also frequently isolated from the roots. We have shown that crop rotation history, soil density, water source and environmental variables, such as precipitation and temperature, within an agricultural ecosystem directly influence the richness and abundance of fungal species colonizing plant roots. The information provided in this study can be used to improve management strategies for soybean seedling pathogens, for example by guiding seed treatment packages and fungicide products to target the most abundant and prevalent known pathogenic species within soybean fields. The potential biocontrol agents identified in this study might also be efficacious against soybean pathogens. The activity of these specific isolates should therefore be explored in future research. Additionally, the fungi incidence and distribution data generated in this study may serve as a benchmark that could be used in future research to monitor changes in the composition of the profiles of the predominant species in these locales due to management practices or changes in environmental conditions.

## CONFLICT OF INTEREST

No conflict of interest declared.

## Supporting information


File S1

Table S1
