# Supplementary material for: Ecology and diversity of culturable fungal species associated with soybean seedling diseases in the Midwestern United States
Source: J Appl Microbiol. 2022 Mar 8;132(5):3797–811. doi: 10.1111/jam.15507 (PMC9311804; doi:10.1111/jam.15507)
Supplement: Supplementary file 2 — Table S1 [file JAM-132-3797-s002.docx]

**Table S1.** Alpha-diversity indexes and evenness from fungal communities isolated from the roots of soybean seedlings collected from different fields within eight Midwestern US states in 2012 and 2013.

|  |  | Abundance | |  | Richness | |  | Shannon-Wiener index | |  | Simpson's index | |  | Evenness^c^ | |
| --- | --- | --- | --- | --- | --- | --- | --- | --- | --- | --- | --- | --- | --- | --- | --- |
| State, year ^a^ | Fields^b^ | Mean | SD |  | Mean | SD |  | Mean | SD |  | Mean | SD |  | Mean | SD |
| Arkansas 2012 | 6 | 14.7 a | ±7.3 |  | 5.8 a | ±2.6 |  | 1.51 a | ±0.41 |  | 0.73 | ±0.10 |  | 0.88 d | ±0.10 |
| Arkansas 2013 | 6 | 32.2 cd | ±9.8 |  | 7.7 ab | ±1.6 |  | 1.55 ab | ±0.28 |  | 0.72 | ±0.08 |  | 0.76 ab | ±0.07 |
| Illinois 2012 | 6 | 27.7 bc | ±2.9 |  | 9.8 bcde | ±2.7 |  | 1.87 ad | ±0.53 |  | 0.76 | ±0.16 |  | 0.82 ad | ±0.14 |
| Illinois 2013 | 6 | 45.3 f | ±3.8 |  | 9.7 bcd | ±1.9 |  | 1.69 ac | ±0.35 |  | 0.73 | ±0.12 |  | 0.74 a | ±0.11 |
| Indiana 2012 | 6 | 24.3 bc | ±3.4 |  | 10.0 bcde | ±2.3 |  | 1.94 cd | ±0.32 |  | 0.79 | ±0.08 |  | 0.85 bcd | ±0.08 |
| Indiana 2013 | 6 | 43.5 f | ±3.4 |  | 13.8 f | ±3.3 |  | 2.05 cd | ±0.38 |  | 0.79 | ±0.09 |  | 0.78 ac | ±0.08 |
| Iowa 2012 | 7 | 22.9 ab | ±7.8 |  | 9.4 bc | ±3.6 |  | 1.85 ad | ±0.38 |  | 0.78 | ±0.07 |  | 0.85 cd | ±0.05 |
| Iowa 2013 | 6 | 45.0 f | ±3.2 |  | 10.5 bcde | ±2.6 |  | 1.82 ad | ±0.29 |  | 0.78 | ±0.06 |  | 0.78 ac | ±0.05 |
| Kansas 2012 | 6 | 15.2 a | ±7.4 |  | 6.3 a | ±1.6 |  | 1.55 ab | ±0.25 |  | 0.73 | ±0.08 |  | 0.86 cd | ±0.08 |
| Kansas 2013 | 5 | 24.8 bc | ±2.4 |  | 8.8 ac | ±1.3 |  | 1.85 ad | ±0.15 |  | 0.80 | ±0.03 |  | 0.85 bcd | ±0.02 |
| Michigan 2012 | 8 | 40.8 ef | ±15.9 |  | 12.6 ef | ±3.9 |  | 2.13 d | ±0.25 |  | 0.84 | ±0.04 |  | 0.86 cd | ±0.03 |
| Michigan 2013 | 6 | 39.2 df | ±6.8 |  | 11.3 cf | ±1.6 |  | 1.89 bcd | ±0.24 |  | 0.76 | ±0.1 |  | 0.79 ac | ±0.10 |
| Minessota 2012 | 6 | 20.5 ab | ±2.3 |  | 8.3 ac | ±2.2 |  | 1.80 ad | ±0.26 |  | 0.79 | ±0.05 |  | 0.86 cd | ±0.03 |
| Minessota 2013 | 6 | 32.8 cde | ±4.3 |  | 11.2 cf | ±3.1 |  | 2.07 d | ±0.35 |  | 0.83 | ±0.06 |  | 0.86 cd | ±0.06 |
| Nebraska 2012 | 5 | 23.8 ac | ±14.8 |  | 8.2 ac | ±2.2 |  | 1.85 ad | ±0.21 |  | 0.82 | ±0.04 |  | 0.90 d | ±0.06 |
| Nebraska 2013 | 6 | 44.2 f | ±5.0 |  | 12.3 df | ±2.7 |  | 2.03 cd | ±0.28 |  | 0.82 | ±0.05 |  | 0.81 ad | ±0.06 |
| ^a^ Data represent state-year average (Mean ± SD) fungal isolates from soybean seedlings across sampled fields | | | | | | | | | | | | | | |  |
| ^b^ Number of fields sampled in each state-year | | | | | | | | | | | | | | |  |
| ^c^ Pielou’s evenness= Shannon-Winer diversity index divided by the natural logarithm of total species in a sample | | | | | | | | | | | | | | | |
| The same letters within each column are not statistically different by LSD p<0.05. | | | | | | | | | | | | | | | |
